# Book Reviews

**Published:** 1896-03

**Authors:** 


### Book Reviews.

The Principles and Practice of Medicine. Designed for the use of Practitioners and Students of Medicine. By William Osler, M. D., Fellow of the Royal College of Physicians, London; Professor of Medicine in the Johns Hopkins University and Physician-in-chief to the Johns Hopkins Hospital, Baltimore, etc., etc. Octavo, pp. xvi.—1,143. Second edition. New York: D. Appleton & Co. 1895.

When Osler's first edition appeared we devoted considerable space to its review, [see p. 754, Vol. XXXI., July, 1892,] and now purpose merely to call attention to changes in the present edition, or to points overlooked in the former notice. The general plan of the work remains the same. The first section is devoted to the consideration of specific infectious diseases, and this is divided into thirty-one subsections, comprising 291 pages.

Typhoid fever, as might be expected, heads the list, and is a subject always fraught with interest. Osler asserts that in cities the prevalence of this disease is directly proportionate to the inefficiency of the drainage and the water supply; and that there is no truer indication of the sanitary condition of a city than the returns of the number of cases of typhoid fever. This has been again and again demonstrated and Buffalo is a striking example of the truism. The hydrotherapy of typhoid receives due attention and Osler asserts that for the fever and its concomitants there is no treatment so efficacious. Directions are given, and substitutes for the bath, when it is impracticable, are suggested. Antipyretics, especially the recent ones, are very properly condemned.

Constitutional diseases occupy section two, which is divided into twelve subsections. Diseases of the digestive system cover the next section, consisting of 161 pages, divided into twelve subsections. The evil effects of mouth-breathing are described in treating of tonsilitis, and the excision of the tonsils is advised when they are large and the general state of health is influenced by them. In the treatment of constipation we miss any allusion to the high enema in the knee-chest posture, that often serves a good purpose in dislodging scybala and large fecal accumulations.

The next section is devoted to the consideration of diseases of the respiratory system, to which about 100 pages are appropriated. Roe, of Rochester, and Daly, of Pittsburg, are credited with the demonstration that hay fever is generally associated with nasal disease. Osler says that in the etiology of hay fever three elements prevail—a nervous constitution, an irritable nasal mucosa and the stimulus. Just what the latter is or may be he does not state.

Following this section comes diseases of the circulatory system, to which, also, about 100 pages are assigned. Osler is quite unsatisfactory in the therapy of these affections, especially of the cardiac neuroses. However, his description is good and in pathology he rises to the occasion. In section six, diseases of the blood and ductless glands are handled with ability, and so, too, are diseases of the kidneys in section seven. The importance of a more complete understanding of these diseases, both as to pathology and treatment, as our civilisation changes and moves further from the primitive, appears to be appreciated.

The next three sections—eight, nine and ten—are devoted to the nervous system, section eight treating of nervous diseases proper, section nine of muscular atrophies, while the intoxications, sunstroke and obesity form the substance of section ten. In section eight, under the head of general introduction, is given a very plain and concise description of the "neuron," and the relation of one neuron to another, enabling the reader to understand fully the latest theory in regard to nerve units. The paths of the different tracts, with their respective neurons, is mapped out clearly, aided by several excellent diagrams, modified after Van Gehuchten. The lesions affecting the cranial nerves and the peripheral nerves are next discussed and the treatment reviewed, embracing the latest suggestions from various writers. The author has, in great part, escaped the adverse criticism of neglecting the "treatment," for all through these chapters the treatment of the various lesions and affections is taken up at greater length than in many of the special works on nervous diseases.

The diseases, acute and chronic, of the spinal cord are discussed in the author's masterly way, embracing the latest theories and pathological findings, especially of the French and German schools. The treatment of locomotor ataxia by anti-specific remedies is deprecated, as it tends to hasten the disease rather than retard or mitigate it, and in this view the author coincides with Gower's opinion. Apparently not much credence is placed upon Gray's reputed cures, as the author has never seen a case of this disease ever cured. The fact is mentioned and is borne out by cases, now being reported the world over, the optic nerve atrophy, one of the most serious events in the disease, has this hopeful aspect—that incoördination rarely follows and the progress of the disease may be arrested. The author states that suspension has been practically abandoned, as the effect seemed to have been more psychical than physical or real.

Part IV., of chapter eight, is devoted to topical diagnosis of diseases of the brain and under the entire description of cerebellar diseases is taken from Dr. W. C. Krauss's paper on the cerebellum, read at the state medical society in 1895.

The chapters on the membrane and blood-vessel lesions of the brain are carefully gone over and receive proper attention; they have of late been slighted in some of the special volumes on nervous diseases. Under general and functional diseases are included acute delirium, chorea, Parkinson's disease, epilepsy, hysteria, and the like. The author commends White's idea of operation, *per se*, in the treatment of epilepsy, but relies mostly on the bromides. The Flechsig, or opium treatment, is not mentioned, the author doubtless having seen no good results follow its use.

Tetany is not considered as an hysterical disease as some of the French writers hold, but is due, according to the author, in many cases to disturbance in the thyroid function, debility, epidemic causes and gastro-intestinal disturbances.

Basedow's disease is placed among the diseases of the thyroid gland—following the theory of Möbius and Greenfield that it is a disease of this gland in antithesis to myxedema. This theory will be sharply contested by the supporters of the medulla and sympathetic nerve theories, with fair prospects of a draw regarding its true etiology.

The last chapter is devoted to alcohol, opium, lead, arsenic, ptomaine and germ poisoning, obesity and sunstroke, and are excellently portrayed. The author has given American writers due attention, and, at the same time, has not slighted foreign authorities. In this respect he differs from the European writers who believe that the subject under discussion is a national one and not international.

The changes have been so numerous in this edition that it becomes almost necessary to discard the former one as a treatise of reference. Not a section has escaped revision, additions, or changes in some respect, many of which are material.

This treatise of Osler will serve to maintain his popularity as a teacher, and it will become further established as a text-book as time advances and its excellent qualities become better understood.

The Year-Book of Treatment for 1896. A Critical Review for Practitioners of Medicine and Surgery. Duodecimo, 484 pages. Cloth, $1.50. Philadelphia: Lea Brothers & Co., Publishers. 1896.

This annual, which has become so popular with many physicians is out with great promptness for the current year. It contains the usual synopsis of accepted methods of treatment on almost every disease that is likely to affect the human race, and presents in easily accessible form the advances made during the past year in the several branches of medicine and surgery. Besides, it presents a classified list of new books of value, medical instruments and surgical appliances, together with pharmaceutical and dietetic novelties, as well as an index of subjects. Taken altogether, it is one of the most complete books of its kind and will find an assured market.

